# Current Trends in Endodontic Clinical Practice in Croatia: A Report From a Croatian National Survey

**DOI:** 10.1002/cre2.70073

**Published:** 2025-01-14

**Authors:** Josipa Sović, Sanja Šegović, Jurica Matijević, Božidar Pavelić, Ivica Anić, Ivan Tomasic

**Affiliations:** ^1^ Postgraduate PhD Study School of Dental Medicine University of Zagreb Zagreb Croatia; ^2^ Dental Clinic of the Health Center Križevci Križevci Croatia; ^3^ Department of Endodontics and Restorative Dentistry School of Dental Medicine University of Zagreb Zagreb Croatia; ^4^ School of Innovation, Design and Engineering Mälardalen University Västerås Sweden

**Keywords:** apex‐locator, endodontics, engine‐driven Instrumentation, root canal obturation, root canal preparation, rubber dam

## Abstract

**Objective:**

To assess the state of endodontic practices and identify factors influencing the use of modern endodontic techniques among dental practitioners in Croatia.

**Materials and Methods:**

A questionnaire was distributed to all dental offices in Croatia, yielding 819 responses (27% response rate). Data were descriptively analyzed and statistically modeled.

**Results:**

Nearly 74% of endodontic treatments in Croatia are performed by general practitioners (17 teeth per month per practitioner), while endodontic specialists handle the highest number of treatments per specialist (67 teeth per month, representing 15% of total treatments). 56% of respondents use radiography before the procedure “always” or “almost always” with intra‐oral periapical radiography being the most commonly used technique. Rubber dams are used “never” to “rarely” by 74% of respondents. Apex‐locators are highly prevalent, used “almost always” to “always” by 83% of practitioners. Magnifiers are rarely used, with 63% of respondents reporting they “never” use them. Lasers are never used by 92% of respondents, whereas 59% frequently use engine‐driven instrumentation. 76% of respondents “always” or “almost always” determine the working length of the root canal. Sodium hypochlorite is the most common irrigant (88%), and EDTA for smear layer removal is used by 36% of respondents. The cold lateral condensation is the most popular obturation technique (64%). CBCT is unused by 59% of responders, most probably because it is not accessible.

**Conclusions:**

Croatian endodontic practices are comparable to those in countries with similar economic status, but there is substantial potential, particularly among general practitioners, to enhance knowledge and awareness of modern technologies through continuous education. Underused techniques in Croatia include rubber dams, magnifiers, lasers, and CBCT.

## 1 Introduction

Standards for endodontic procedures include the use of rubber dams for isolation, apex‐locators for working length determination, and sodium hypochlorite for canal irrigation (Duncan et al. 2023, 2021; Eliyas et al. 2018; Löst 2006). Other important variables for the quality of endodontic treatment (ET) include planning, patient medical and dental history, radiographic analysis, preparation of the endodontic access cavity, root canal instrumentation, disinfection, irrigation, smear layer removal, obturation, magnification, and post‐ET recalls (Azim, Merdad, and Peters 2022; Cheung, Peters, and Parashos 2023).

Clinical endodontic practice has been investigated in numerous countries such as the United States (Eleazer et al. 2016; Savani et al. 2014; Whitten et al. 1996), Denmark (Bjørndal and Reit 2005; Markvart, Fransson, and Bjørndal 2018; Sørensen and Kirkevang 2021), New Zealand (Tay et al. 2008; van der Zande et al. 2018), Turkey (Gul Celik Unal, Tac, and Kececi 2012; Kaptan et al. 2012; Topkara et al. 2017), and among Flemish dentists (Hommez, De Moor, and Braem 2003; Neukermans et al. 2015; Slaus and Bottenberg 2002). Research has focused on topics such as the adaptation of novel technologies like engine‐driven instruments (Awk Chan, Cheung, and Rpy 2006; Parashos and Messer 2004) and CBCT (Krug et al. 2019; Setzer et al. 2017). Other areas of focus include single‐visit versus multiple‐visit treatments (Bird, Chambers, and Peters 2009; Inamoto et al. 2002; Logsdon et al. 2020; Sathorn, Parashos, and Messer 2009), regenerative endodontic procedures (Epelman et al. 2009; Lee et al. 2018), the use of dental operating microscopes (Kersten, Mines, and Sweet 2008; Mines et al. 1999), disinfection protocols (Clarkson et al. 2003; de Gregorio et al. 2015; Dutner, Mines, and Anderson 2012), and access cavity and instrumentation techniques (Tsotsis et al. 2021).

Previous research on dental practice in Croatia has explored medication prescribing (Šutej et al. 2021, 2023), antibiotic administration (Šimundić Munitić et al. 2021; Perić et al. 2015; Sović et al. 2020, 2024b), and the referral of problematic endodontically treated teeth to oral surgery (Babić et al. 2019). Other studies have examined medical emergencies in dental practice (Firić 2022), emergency procedures for dental trauma among pediatricians (Nikolic et al. 2018), and occupational injuries among Croatian dentists (Savić Pavičin et al. 2020). Further research has investigated dental students' knowledge of dental care for oncology patients (Pedic et al. 2021) and the need for improved dental record maintenance for forensic purposes (Savić Pavičin et al. 2021). Given that undergraduate training focused on manual canal instrumentation using the step‐back technique was only recently supplemented with engine‐driven instruments and techniques, it is not a surprise that its adaptation has not yet been investigated.

This study investigates endodontic clinical practice in Croatia and compares it to global practices. The primary goal is to identify areas for improvement in continuous professional development and to gather evidence for influencing governmental policies on endodontic treatments. The secondary objective is to investigate the adaptation of engine‐driven instrumentation among Croatian dentists.

## 2 Materials and Methods

This study is part of the dissertation titled “Assessment of Procedures in the Performance of Endodontic Therapy in Dental Offices in the Republic of Croatia,” (no. 05‐PA‐24‐3/2018) approved by the Ethics Committee of the University of Zagreb.

### 2.1 Questionnaire

The questionnaire was sent to an estimated 3000 dental practitioners in Croatia. The number of responders was estimated using data from the Croatian Institute of Public Health and the Croatian Health Statistics yearbook. The questionnaire is available at https://forms.gle/nmUeQizSoN2U5SNYA.

Participants provided demographic information, including sex (female – F, male – M), years of practice, educational qualification (DDM – Doctor of Dental Medicine, EndoS – Specialist in Endodontics, EndoR – Resident in Endodontics, Other), type of dental office (HC – Health Center, InConc – Dental Clinic with Concession Contract, Priv – Private Clinic, Priva SA – Private Clinic with a Health Fund Contract, Poly – Dental Polyclinic, SDM – School of Dental Medicine), and continuous education in endodontics taken in the last 5 years (CE). The questionnaire included questions on endodontic clinical practices, such as:

- Number of endodontically treated teeth per month.
- Use of rubber dams and apex locators.
- Use of magnifiers, XR images, and CBCT.
- Manual versus engine‐driven versus combined instrumentation.
- Irrigation, use of chelators, ultrasound, final irrigation, use of lasers.
- Obturation technique, use of spreaders, and more.

The questionnaire also included a field for the subjective evaluation of endodontic practice and an open field for free text comments.

### 2.2 Statistical Methods

Analysis was performed using R Project for Statistical Computing (version 4.3.0) with the Survey package to account for finite population correction. The distribution of practitioners by gender, years of practice, and CE was examined. The $\chi^{2}$ test assessed relationships between categorical variables, while ANOVA was used for continuous and categorical variables. Logistic and ordinal regression models were constructed and adjusted for gender and years of practice. Effects are reported as log odds (LO), with significance codes: ***0.001, **0.01, *0.05, 0.1. Slopes are designated with a corresponding number of stripes whereas a dotted stripe corresponds to a dot, i.e., to 0.1 significance.

## 3 Results

### 3.1 Characteristics of Practitioners

By June 2023, 819 responses were collected, representing a 27% response rate. After excluding 31 responders who do not perform ETs (3.8%), the analysis focuses on those actively engaged in endodontics.

### 3.2 General Description

Table 1 displays the number of endodontically treated teeth per month by degree of clinical education (DCE), whereas Table 2 summarizes general numbers related to endodontic practice. Almost 74% of ETs in Croatia are performed by DDMs, with each treating an average of 17 teeth per month. Among specialists, EndoSs manage the highest number of treatments per practitioner (67 teeth per month, 15% of total treatments). CE correlates with increased number of treated teeth, with a factor of 6.3 (*p* = 0.001).

**Table 1:** Number of teeth per month per DCE.

DCE	Number of teeth (SE)	Number of teeth (%)
Doctor of Dental Medicine	17 (0.5)	73.9%
Specialist in endodontics	67 (6.8)	15%
Resident in endodontics	37 (4.2)	5%
Other	17 (1.5)	6.1%

**Table 2:** Endodontic practice.

	% (SE)
**Number of ET teeth per month**	
< =5	11.8%
6–10	30.2%
11–20	36.4%
21–30	9.3%
> 30	12.3%
**Use of rubber dam**	
Never	43.3% (0.02)
Very rarely	13.4% (0.01)
Rarely	17.2% (0.01)
Often	11.6% (0.01)
Almost always	6.8% (0.01)
Always	7.9% (0.01)
**Use of magnifier**	
Never	62.7% (0.01)
Very rarely	8.6% (0.01)
Rarely	8.9% (0.01)
Often	8.5% (0.01)
Almost always	3.3% (0.01)
Always	8.1% (0.01)
**Determining the canal length**	
Never–very rarely	1.5% (0)
Rarely	2.1% (0)
Often	20.2% (0.01)
Almost always	9.9% (0.01)
Always	66.3% (0.01)
**Working length determination method**	
Apex‐locator	93.4%
XR image	29.7%
XR image with an instrument or a gutta‐percha stick of a known length	21%
By the feel of the instrument in the hand	20%
Until the patient feels a stab with an instr.	15.4%
Other	0.6%
**Use of apex‐locator**	
Never	4.3% (0.01)
Very rarely	1.8% (0)
Rarely	2.7% (0.01)
Often	7.8% (0.01)
Almost always	8.9% (0.01)
Always	74.5% (0.01)
**Use of engine‐driven instrumentation**	
Never	27.3% (0.01)
Very rarely	6.1% (0.01)
Rarely	7.7% (0.01)
Often	27% (0.01)
Almost always	17.4% (0.01)
Always	14.5% (0.01)
**Use of engine‐driven and manual instrumentation**	
Never	27.5% (0.01)
Very rarely	7.3% (0.01)
Rarely	8.9% (0.01)
Often	26.1% (0.01)
Almost always	12.2% (0.01)
Always	18% (0.01)
**Manual instrumentation**	
Never	2.3% (0)
Very rarely	11.6% (0.01)
Rarely	14.1% (0.01)
Often	29.1% (0.01)
Almost always	12.8% (0.01)
Always	30.1% (0.01)
**Chelation**	
Never	4.3% (0.01)
Very rarely	6.5% (0.01)
Rarely	14.1% (0.01)
Often	40.2% (0.02)
Almost always	11.7% (0.01)
Always	23.4% (0.01)
**Use of laser**	
Never	91.7% (0.01)
Very rarely	1.7% (0)
Rarely	2.5% (0)
Often	3.5% (0.01)
Almost always–Always	0.5% (0)
**Use of ultrasound**	
Never	72.3% (0.01)
Very rarely	6.5% (0.01)
Rarely	8.5% (0.01)
Often	8.7% (0.01)
Almost always	2.8% (0.01)
Always	1.3% (0)
**Final irrigation**	
Never–Very rarely	2.3% (0)
Rarely	2.9% (0.01)
Often	25.7% (0.01)
Almost always	15.3% (0.01)
Always	53.8% (0.02)
**Use of spreaders**	
Never	7.6% (0.01)
Very rarely	3.9% (0.01)
Rarely	10.1% (0.01)
Often	27% (0.01)
Almost always	12.3% (0.01)
Always	39.2% (0.02)
**Use of CBCT**	
Never	58.9% (0.02)
Very rarely	13% (0.01)
Rarely	16.8% (0.01)
Often	11.3% (0.01)

### 3.3 Endodontic Practices

General characteristics of practitioners are presented in Figure 1 while key parameters influenced by practice are illustrated in Figures 2, 3, 4, 5, 6, 7, 8, 9, 10, 11, 12, 13, 14, 15, 16, 17.

**Figure 1:**
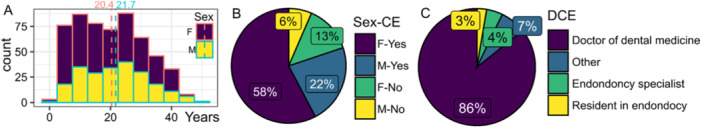
General characteristics of practitioners. (A) Years of practice; (B) attended continuous education in the last 5 years (CE); (C) degree of clinical education (DCE).

**Figure 2:**
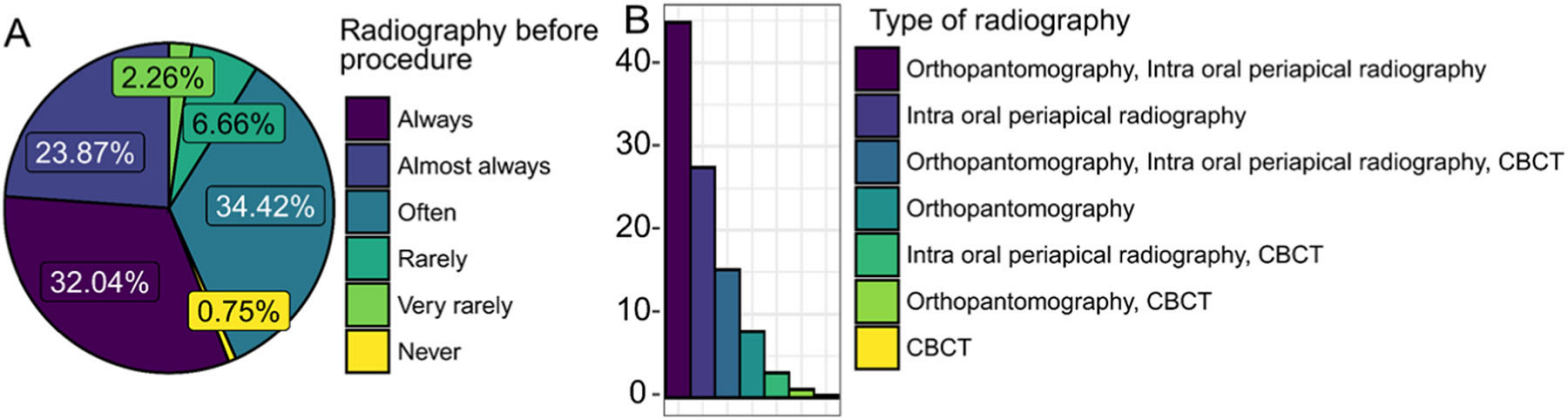
Radiography before procedure.

**Figure 3:**
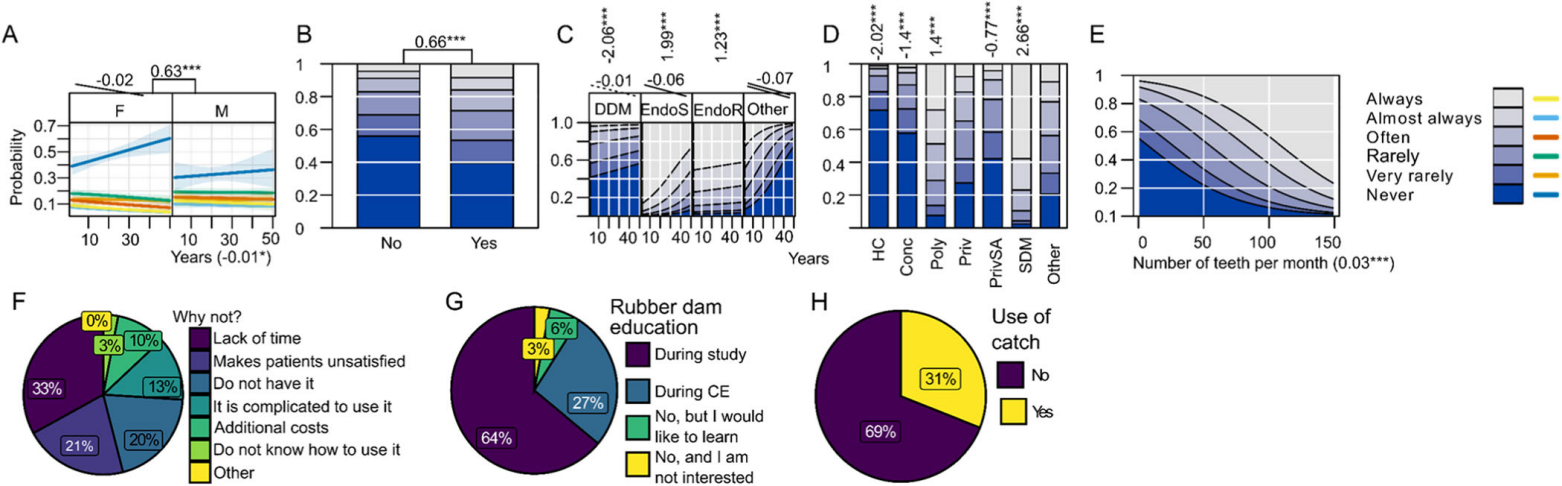
Rubber dam. (A) Effects of years of practice and sex; (B) attended continuous education in the last 5 years (CE); (C) degree of clinical education (DCE); (D) office organization; (E) number of teeth; (F) why not using it; (G) education; (H) use of protection for the clamp.

**Figure 4:**
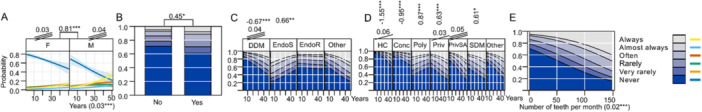
Magnifier. (A) Effects of sex and years of practice; (B) attended continuous education in the last 5 years (CE); (C) degree of clinical education (DCE); (D) office organization; (E) number of teeth.

**Figure 5:**
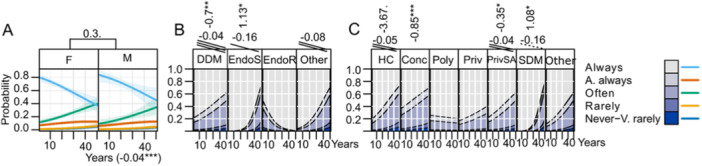
Working length determination. (A) Sex and years of practice; (B) degree of clinical education (DCE); (C) office organization.

**Figure 6:**
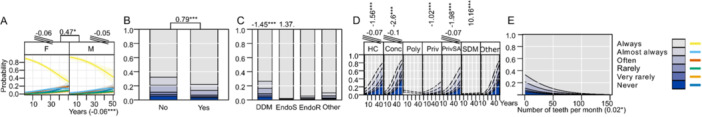
Apex‐locator. (A) Effect of sex and years of practice; (B) attended continuous education in the last 5 years (CE); (C) degree of clinical education (DCE); (D) office organization; (E) number of teeth.

**Figure 7:**
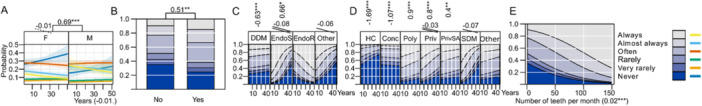
Engine‐driven instrumentation. (A) Effect of sex and years of practice; (B) attended continuous education in the last 5 years (CE); (C) degree of clinical education (DCE); (D) office type; (E) number of teeth.

**Figure 8:**
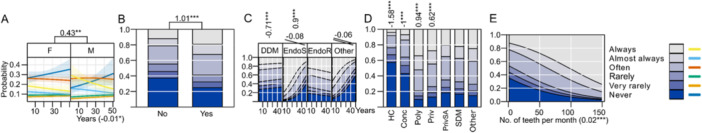
Combination of engine‐driven and manual instrumentation. (A) Effect of sex and years of practice; (B) attended continuous education in the last 5 years (CE); (C) degree of clinical education (DCE); (D) office type; (E) number of teeth.

**Figure 9:**
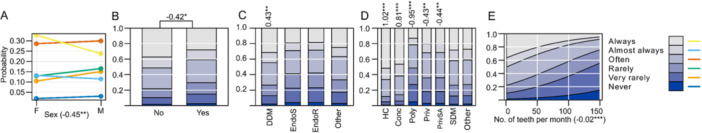
Manual instrumentation. (A) Effect of sex; (B) attended continuous education in the last 5 years (CE); (C) degree of clinical education (DCE); (D) office type; (E) number of teeth.

**Figure 10:**
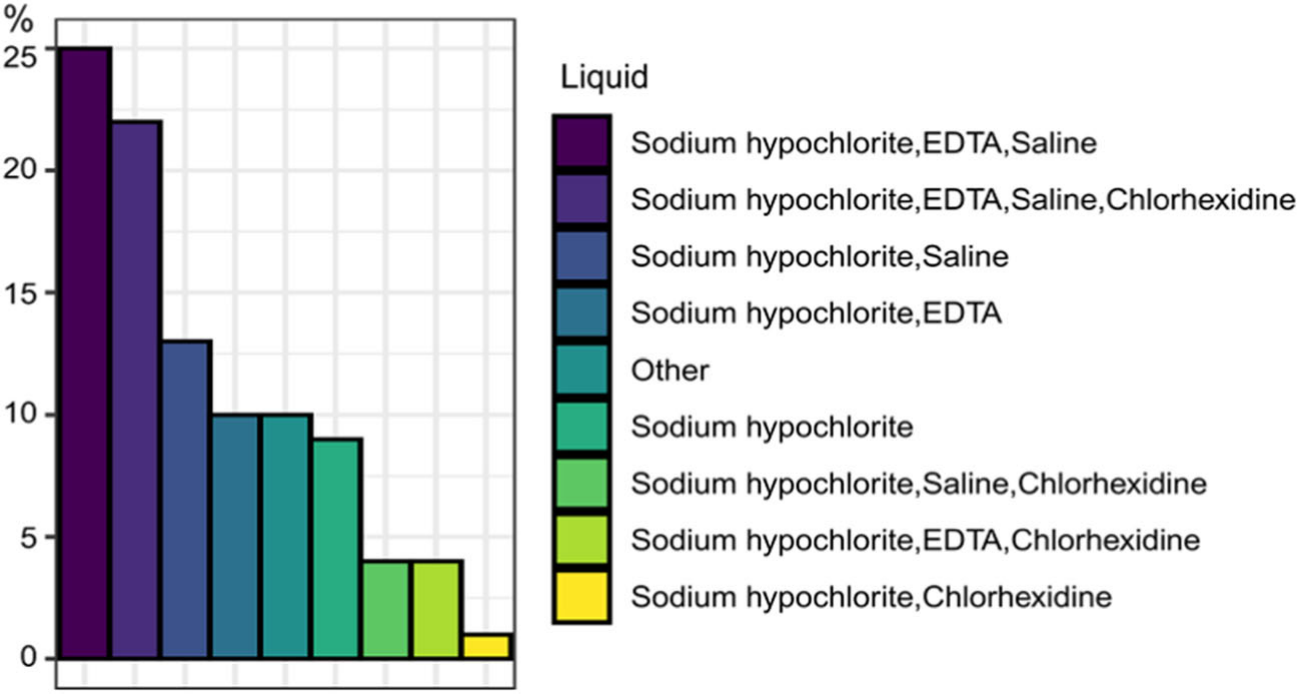
Irrigation.

**Figure 11:**
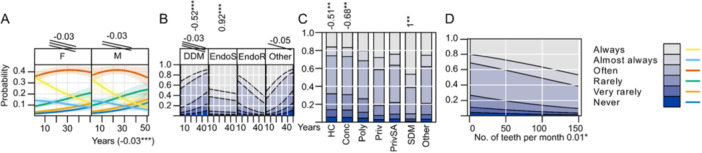
Chelation. (A) Sex and years of practice; (B) degree of clinical education (DCE); (C) office; (D) number of teeth.

**Figure 12:**
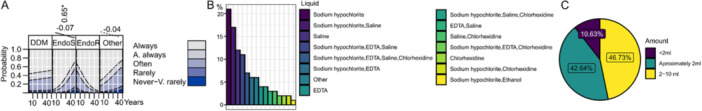
Final irrigation for smear layer removal. (A) Degree of clinical education (DCE); (B) irrigants; (C) amounts of irrigants.

**Figure 13:**
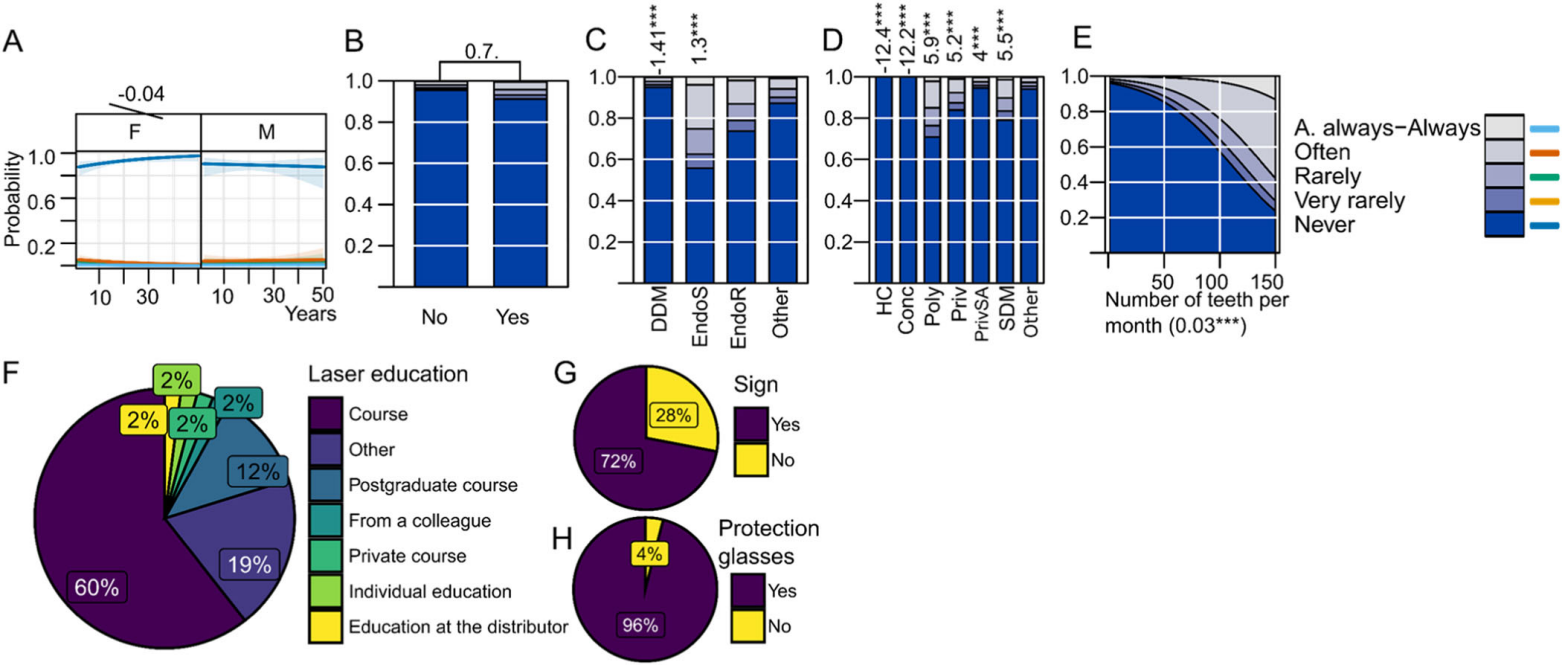
Laser. (A) Effect of sex and years of practice; (B) attended continuous education in the last 5 years (CE); (C) degree of clinical education (DCE); (D) office organization; (E) number of teeth; (F) continuous education for laser; (G) warning sign at the entrance; (H) protection glasses.

**Figure 14:**
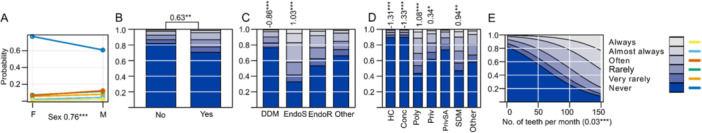
Use of ultrasound instruments. (A) Sex; (B) attended continuous education in the last 5 years (CE); (C) degree of clinical education (DCE); (D) office organization; (E) number of teeth.

**Figure 15:**
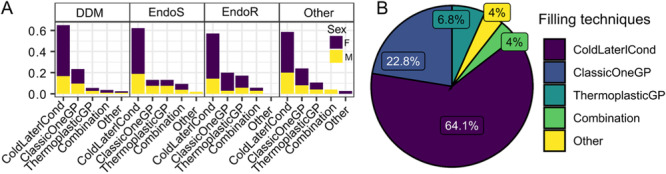
(A) Obturation by degree of clinical education (DCE) and sex. Percentages are within each DCE. (B) Percentage for each filling technique.

**Figure 16:**
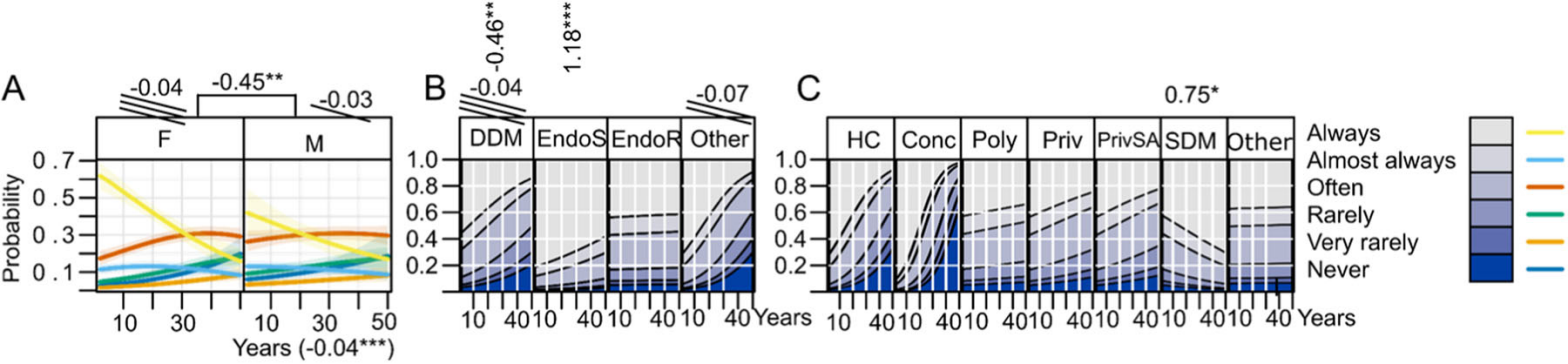
Use of spreaders. (A) Sex and years of practice; (B) degree of clinical education (DCE); (C) office organization.

**Figure 17:**
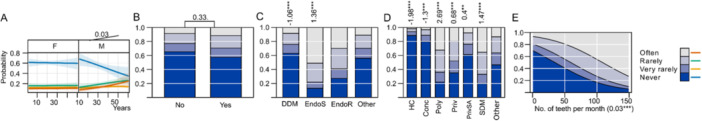
Use of CBCT. (A) Effect of sex and years of practice; (B) CE; (C) degree of clinical education (DCE); (D) office organization; (E) number of teeth per month.

Figure 2 shows that more than half of respondents *always* or *almost always* use some form of **preoperative radiography**. The most commonly used technique is intra oral periapical radiography, with 27.5% of respondents using it exclusively and an additional 63.2% using it in combination with other methods. Only 7.9% of respondents use orthopantomography exclusively.

**Rubber dams** are infrequently used, with 74% of practitioners reporting they *never* to *rarely* use them. Usage decreases with years of practice (LO = −0.01*), increases with CE (LO = 0.66***), and is positively associated with the number of teeth treated per month (LO = 0.03***) (Figure 3). Figure 3F–H show the primary reasons for non‐use include lack of time and patient discomfort; most practitioners acquired rubber dam education during their university but 69% do not protect the clamp during application to prevent accidental swallowing.

**Magnifiers** are rarely used, with 63% of respondents reporting they *never* use them. Magnifier usage increases with CE (LO = 0.45*), the number of teeth treated per month (LO = 0.02***), and years of practice (LO = 0.03***). DDMs use it less (LO = −0.67***) as well as HC (LO = −1.55***) and Conc (LO = −0.95***) offices (Figure 4).

**Working length determination** is standard practice, with 76.2% of practitioners *always* or *almost always* measuring it (Table 2). Endos slightly exceed others in this practice (LO = 1.13*), whereas HC (LO = −3.67.), Conc (LO = −0.85***), and PrivSA (LO = −0.35*) are offices in which it is significantly less practiced. (Figure 5). Apex locators are the most common tool (93%), complemented with XR images, tactile feedback, or even measuring until patient sensation occurs (Table 2). **Apex‐locator** usage is high (83% use it *always* or *almost always*) but decreases with years of practice (LO = −0.06***). CE correlates positively with higher usage (LO = 0.79***). EndoS uses apex locators significantly more (LO = 1.37), while DDM use them less (LO = −1.45***) which is reflected by the fact that SDM uses it significantly more (LO = 10.16***) and HC significantly less (LO = −1.56***) since it is in SDM where EndoS are mostly employed and HCs are mostly populated with DDMs. Apex‐locator usage increases with each number of teeth treated per month (LO = 0.02*) (Figure 6).

**Engine‐driven instrumentation** is common, with 59% of respondents using it *often* to *always*. EndoS use it more often (LO = 0.66*), while DDMs tend to favor manual instrumentation (LO = 0.43**). CE has a positive impact on engine‐driven instrumentation adaptation (LO = 0.51**), whereas manual instrumentation correlates negatively with CE (LO = −0.42*) (Figures 7 and 9). Practitioners using a combination of techniques (Figure 8) are primarily EndoS (LO = 0.9***) and those with CE (LO = 1.01***).

Twenty‐five percent of respondents reported using three **irrigants**—sodium hypochlorite, ethylenediaminetetraacetic acid (EDTA), and saline—while 22% included chlorhexidine as a fourth irrigant (Figure 10). When analyzing each irrigant individually, sodium hypochlorite was the most commonly used (88%), followed by saline (64%), EDTA (61%), and chlorhexidine (33%).

**Chelator** usage is common (Table 2), but lower among general practitioners (LO = −0.52***) (Figure 11). Calcinasa by Lege Artis Pharma (77.5%) and Glyde by Dentsply (11.8%) are the most popular chelators.

Final irrigation for **smear layer removal** is *always* or *almost always* performed by 69.1% of respondents, with EndoS performing it significantly more (LO = 0.65*) (Figure 12). The most commonly used final irrigant is sodium hypochlorite, either alone (20.7%) or in combination with other solutions (Figure 12B). Additionally, 12% of respondents reported relying exclusively on saline for final irrigation. Notably, approximately 90% of practitioners use a minimum of 2 mL of irrigant per application (Figure 12C). Chlorhexidine is used by 20.1% of respondents. EDTA was reported by only 36.2% of respondents, with 81.3% of those practitioners having completed CE and averaging 21 years of practice (std. error (SE) = 0.65).

**Laser** technology is rarely used, with 91.7% of respondents never using lasers. CE has a minor positive impact (LO = 0.7), but laser adoption remains low with only EndoS using it to some degree (LO = 1.3***) (Figure 13). Safety protocols need improvement, with 28% of laser users omitting warning signs and 4% not using protective glasses. Figure 13F shows that 60% of practitioners got laser training on a course.

**Ultrasound** is similarly underused but positively influenced by CE (LO = 0.63**) and favored by EndoS (LO = 1.03***) (Figure 14).

Cold lateral condensation remains the most common **obturation** technique (64.1%) for all practitioners regardless of DCE (Figure 15).

**Spreader** usage decreases with years of practice (LO = −0.04***), especially among DDMs who use them less than other DCEs (LO = −0.46**), whereas EndoS use spreaders more frequently (LO = 1.18***) (Figure 16).

**CBCT** usage is low, with 59% of respondents *never* using it (Table 2). 56.5% reported they did not have access to a CBCT device. The usage however increases with the number of endodontic treatments performed (LO = 0.03***) and correlates positively with CE (LO = 0.33). EndoS are the most frequent users (LO = 1.36***) with 50% reporting *often* using it (Figure 17).

## 4 Discussion

The number of responses collected (819) is satisfactory compared to a similar global survey within specialist endodontic practice, which garnered 543 responses (Cheung, Peters, and Parashos 2023). Respondents were unevenly distributed by years of experience (Figure 1A), with a higher proportion of females (F) (69.2%) than males (M) (30.8%). Both genders reported comparable mean years of practice (M = 21.7, F = 20.4). In contrast, a US study found that 75% of respondents were male, with 56% having over 20 years of experience (Savani et al. 2014). Moreover, 80% of Croatian respondents had completed CE in endodontics, which has been investigated in more detail by another recent study (Sović et al. 2024a).

**Preoperative radiography** that captures the full roots and at least 2–3 mm of the periapical region is mandatory before initiating root canal treatment (Löst 2006), as it is crucial for accurate diagnosis and treatment planning. While an orthopantomogram serves as a useful initial diagnostic tool, an intraoral periapical radiograph offers a more detailed evaluation of the anatomy and the condition of the teeth and periapical tissues. A CBCT image provides even more comprehensive information by offering a three‐dimensional analysis.

With 55.9% of respondents *almost always* or *always* analyzing radiographic images before ET and an additional 34% doing this often, it is evident that there is room for improvement in this practice. Considering that 90.7% of respondents use intraoral periapical imaging alone or in combination with other radiographic methods, further education could help enhance the effective use of preoperative imaging techniques in clinical practice.

It is widely accepted that the use of **rubber dams** significantly improves the success rate of ETs (Lin et al. 2014). However, in this study, 74% of Croatian respondents reported using a rubber dam *never* to *rarely* even though it has been part of undergraduate education for over 30 years and is mandatory during ETs. In comparison, 60% of US dentists always use rubber dams (Savani et al. 2014), while in India, 48% of practitioners never use rubber dams, and only 23% always use them in the state of Odisha (Mohanty et al. 2023; Sasirekha et al. 2014). In South Africa, 37% of practitioners routinely use rubber dams (Buchanan et al. 2019), whereas in Sweden and Norway, the rate is as high as 96.9% (Malmberg, Hägg, and Björkner 2019). The low use of rubber dams in Croatia is surprising, given the relatively low cost of the equipment. However, it is encouraging that rubber dam usage increases with the number of teeth treated, indicating that clinicians handling more cases prioritize aseptic conditions. While Savani et al. (2014) found no significant association between rubber dam use and CE in the USA, our study found a positive correlation between CE and rubber dam use in Croatia, similar to South Africa (Buchanan et al. 2019).

The main reasons for not using rubber dams in India include patient discomfort, time consumption, application difficulties, lack of training, and financial constraints (Mohanty et al. 2023). In Croatia, the reasons are similar, with some additional comments such as “Destructed tooth is difficult to catch,” “Bad habit,” and “There are so many patients in health centers that I'm just proud I still do endodontics.” Additionally, only 31% of Croatian respondents protect the clamp during rubber dam application to prevent accidental swallowing, which raises safety concerns.

Since the 1970s, **magnification devices** such as microscopes and loupes have been widely used in dentistry due to their clinical benefits (Bud et al. 2021). However, 80.1% of Croatian respondents reported *rarely*, *very rarely*, or *never* using magnifiers, likely due to their cost and the personalized nature of the equipment, which prevents them from being shared in clinics. Some practitioners may also be hesitant to use magnifiers due to concerns about their influence on eyesight. The use of magnifiers tends to increase with years of practice, and men tend to use them slightly more than women. DDMs use magnifiers significantly less than other groups, likely due to stricter budget constraints (Low et al. 2019). Globally, magnification devices are much more widely used with 75% of US dentists routinely using some form of magnification (Savani et al. 2014). Within specialist practice in developed countries, 91.3% of responders reported using dental operating microscopes (Cheung, Peters, and Parashos 2023) compared to 42.9% of EndoS in Croatia reporting using magnification devices *often* to *always*. CE positively correlates with magnifier use in Croatia.

Apex‐locator adaptation in Croatia is high, with nearly 75% of respondents always using an apex‐locator, and 97.1% of EndoS use it *always*, aligning with 99.1% usage among endodontists internationally (Cheung, Peters, and Parashos 2023). Its usage is strongly associated with CE, suggesting further training could enhance adoption.

**Engine‐driven instrumentation** is used frequently by 59% of Croatian respondents, with higher rates among specialists (85.7%) compared to DDMs who tend to rely more on manual instrumentation, possibly due to financial constraints. In developed countries, engine‐driven instrumentation is used by over 95% of endodontists (Cheung, Peters, and Parashos 2023). In India, 46% of EndoS use engine‐driven instruments, while a combination of hand and engine‐driven instrumentation is also common (Mohanty et al. 2023). These numbers indicate that even though postgraduate courses in engine‐driven instrumentation have been available, Croatia still has room for improvement in terms of engine‐driven instrumentation adoption, particularly among general practitioners.

The most often used **irrigant** is sodium hypochlorite with almost all practitioners using it, either alone or in combination with other irritants, mirroring global trends (Buchanan et al. 2019, Savani et al. 2014).

Buchanan et al. (2019) report viscous **chelators** routinely used in 86% of cases whereas in the USA the percentage is 83% (Savani et al. 2014). The percentage of using chelation regularly in Croatia is 75.3%.

Considering that 69.1% of respondents reported *almost always* or *always* performing final irrigation for **smear layer removal**, yet only 36.2% use EDTA and 12% rely solely on saline, it is evident that many practitioners lack a clear understanding of the purpose of final irrigation. EDTA is essential as a rinse for effectively removing the smear layer, while saline is unsuitable as a primary irrigant because it lacks both tissue‐dissolving and antimicrobial properties (Haapasalo et al. 2014). Additionally, the use of chlorhexidine—an irrigant effective against Enterococcus faecalis, a common pathogen in asymptomatic persistent endodontic infections (Wassel, Radwan, and Elghazawy 2023; Wolters et al. 2017)—is notably low, with only 20.1% of respondents reporting its use either alone or in combination with other solutions.

In recent years, new techniques such as high‐power **lasers** and antimicrobial photodynamic therapy have been added as alternative therapy modalities (Bordea et al. 2020; Asnaashari, Sadeghian, and Hazrati 2022). Despite their potential benefits, our survey shows that 92% of respondents never use lasers and CE has a modest but positive impact on the adoption of lasers. When examining laser usage by specialization, EndoR and EndoS are the primary users of this technology. The low adoption of laser technology may be attributed to its relatively high cost, the learning curve associated with its implementation, or the perception that traditional techniques, such as engine‐driven instrumentation and chemical irrigation, remain more cost‐effective or accessible. Moreover, the integration of lasers into daily endodontic practice may depend heavily on the practitioner's access to specialized training, which could explain the higher usage among those with advanced education and within certain institutional settings. Encouraging more widespread education on the potential benefits of laser treatments could help bridge this gap and increase adoption rates among general practitioners as well, at least for those who work in offices equipped with a laser.

The most common **obturation** technique among Croatian dentists (Figure 15) is cold lateral condensation 64.1%, which is not surprising considering that it is the most used and learned technique during undergraduate teaching. Spreaders, as an indispensable auxiliary instrument in the cold lateral condensation technique, are *almost always* or *always* used by only 51.5% of our respondents. In second place, with a total of 22.6% usage is the classic technique with single GP. Among EndoS and EndoR, thermoplacticGP is approximately equally popular as the classic guttapercha point technique. Savani et al. report cold lateral compaction to be the most often used (40%) (Savani et al. 2014), whereas (Buchanan et al. 2019) reported single cone technique at 58%, followed by cold lateral condensation at 23% and as most popular warm techniques vertical condensation 10% followed by carrier‐based obturation 6% in South Africa. The dominant usage of single cone is also confirmed by the study of (Mohanty et al. 2020, 2023).

CBCT usage is low in Croatia, varying significantly by clinic type and the number of teeth treated. It is only used to some extent by EndoS and EndoR with about 50% of EndoS reporting to *often* use it. This is still below the 90% reported among specialists in developed countries (Cheung, Peters, and Parashos 2023) Encouragingly, CBCT usage increases with CE and the number of teeth treated. The main reason for not using CBCT appears to be its inaccessibility therefore there is ample opportunity to expand the use of this technology in Croatia.

**Figure 18:**
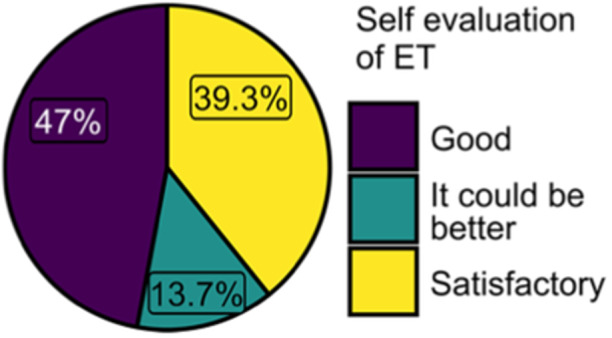
Self‐evaluation of endodontic treatments.

In terms of subjective evaluations of ETs, 47% of Croatian respondents rated their treatments as good (Figure 18), which is in line with findings from a Norwegian and Swedish study, where nearly half of the respondents estimated their endodontic success rate to be 90% (Malmberg et al. 2020). However, the low use of key technologies like rubber dams and CBCT suggests that these self‐assessments may be overly optimistic. Studies from other countries, such as the Netherlands, indicate that about 40% of root‐filled teeth are associated with apical radiolucency, suggesting treatment failure (Wu, Shemesh, and Wesselink 2009, Peters et al. 2011, Love and Firth 2009; Nair et al. 1999). The actual failure rate of ETs done by DDMs can be even higher than that considering that endodontically treated teeth without visible radiographic signs of apical periodontitis can still be infected (Molander et al. 1998; Ricucci, Loghin, and Siqueira 2014). Given that 74% of ETs in Croatia are performed by DDMs, many teeth treated in general practice are probably left inadequately cleaned or filled, leading to retreatments and unnecessary increases in dental care expenses. Therefore, future steps should focus on identifying Croatian regions in which CE should be intensified and on evaluating the frequency and causes of retreatments statistically.

The limitations of this study should be acknowledged. Since the questionnaire allowed for multiple answers to the questions about irrigants without asking for further details, it is not possible to analyze the use of combinations of irrigants, their sequence, as well as the respondents' knowledge of the advantages or disadvantages of using different combinations. One general limitation is the difficulty in comparing this study with others globally due to the lack of standardized questionnaires. Developing a harmonized questionnaire for international studies would greatly enhance future comparisons and facilitate efforts to align endodontic practices across countries. Additionally, as this study is based on a questionnaire, the responses are subjective and cannot directly correlate practices with clinical outcomes showing a need for objective outcome studies to complement the questionnaire‐based findings. Finally, questionnaire‐based studies are subject to response biases in terms of overreporting adherence to best practices or underreporting challenges.

## 5 Conclusion

The implementation and use of modern technologies in Croatian endodontic practice are correlated with the country's living standards and continuous education (CE). Except for regular usage of preoperative intra‐oral periapical radiography and the widespread use of apex locators, there is significant potential, particularly among DDMs, to increase knowledge and awareness of modern technologies through CE. The results also suggest that many unnecessary retreatments may occur due to inadequate initial treatments.

Encouragingly, practitioners who treat more teeth per month tend to be better equipped and are more likely to have CE and use standard techniques such as rubber dams, apex‐locators, engine‐driven instrumentation, and magnifiers more frequently. Expanding access to CE and CBCT can further promote best practices and improve treatment quality, reducing the need for retreatments caused by inadequate initial care.

## Author Contributions

Josipa Sović actively participated in designing the study and the questionnaire explored the related literature, and this study is within her doctoral dissertation. Sanja Šegović actively participated in designing the study and the questionnaire, and wrote the paper. Jurica Matijević was involved in consultations about the study and wrote the paper. Božidar Paveli participated in the interpretation of the results and consultations about the study. Ivica Anić participated in designing the research and questionnaire. Ivan Tomasic analyzed the data and interpreted the results of statistical analysis, wrote the paper, participated in designing the study, and coordinated the study.

## Ethics Statement

This study is a part of the dissertation: “Assessment of Procedures in the Performance of Endodontic Therapy in dental offices in the Republic of Croatia” (no. 05‐PA‐24‐3/2018), which was approved by The Ethics Committee of the University of Zagreb.

## Conflicts of Interest

The authors declare no conflicts of interest.

## Data Availability

The data that support the findings of this study should become available on request from the corresponding author. At the moment the authors are not sure if they are allowed to share the data due to ethical/privacy restrictions.
